# Specificity and regulation of phosphotyrosine signaling through SH2 domains

**DOI:** 10.1016/j.yjsbx.2020.100026

**Published:** 2020-05-27

**Authors:** Michelangelo Marasco, Teresa Carlomagno

**Affiliations:** a Leibniz University Hannover, Institute of Organic Chemistry and Centre for Biomolecular Drug Research, Schneiderberg 38, 30167 Hannover, Germany; b Helmholtz Centre for Infection Research, Group of Structural Chemistry, Inhoffenstrasse 7, 38124 Braunschweig, Germany

**Keywords:** SH2 domain, Phosphotyrosine, Binding specificity, pY signalling

## Abstract

Phosphotyrosine (pY) signaling is instrumental to numerous cellular processes. pY recognition occurs through specialized protein modules, among which the Src-homology 2 (SH2) domain is the most common. SH2 domains are small protein modules with an invariant fold, and are present in more than a hundred proteins with different function. Here we ask the question of how such a structurally conserved, small protein domain can recognize distinct phosphopeptides with the breath of binding affinity, specificity and kinetic parameters necessary for proper control of pY-dependent signaling and rapid cellular response. We review the current knowledge on structure, thermodynamics and kinetics of SH2–phosphopeptide complexes and conclude that selective phosphopeptide recognition is governed by both structure and dynamics of the SH2 domain, as well as by the kinetics of the binding events. Further studies on the thermodynamic and kinetic properties of SH2–phosphopeptide complexes, beyond their structure, are required to understand signaling regulation.

## Highlights

• By specifically recognizing phosphotyrosine-containing motifs, SH2 domains modulate a variety of essential cellular processes.
• Despite their very conserved fold, SH2 domains have achieved a high level of specificity, while ensuring fast response times to changing cellular conditions.
• The conventional view of cellular signaling centered around enthalpy-based equilibrium descriptors cannot account for such a degree of specificity.
• Only an integrated view of structural biology, thermodynamics, binding kinetics and protein dynamics can successfully address the specificity mechanisms of SH2 domains.

## Graphical abstract

**Figure:**
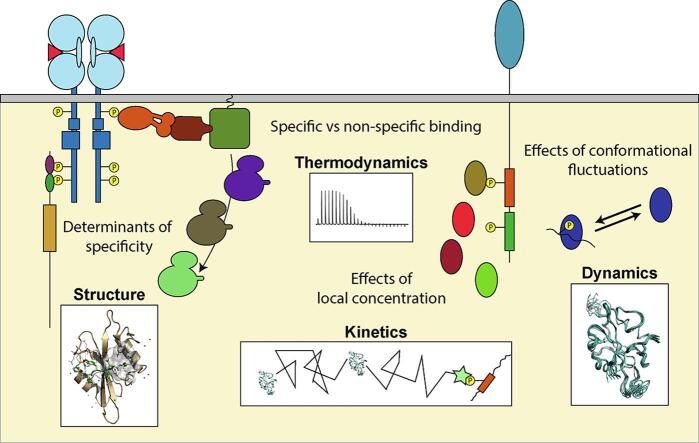

## 1 Introduction

Eukaryotic cells use phosphorylation of tyrosine residues as one of the major routes of regulation of vital processes such as growth, survival, cytoskeletal reorganization, adhesion, metabolic homeostasis, and others (Hunter, 2009). The abundance of the phosphotyrosine mark (pY) is regulated by the opposing activities of protein tyrosine kinases (PTKs), which catalyze the transfer of the γ-phosphate of ATP to the hydroxyl group of tyrosine, and protein tyrosine phosphatases (PTPs), which hydrolyze the phosphoester bond of phosphotyrosine. Next to tyrosine, serine and threonine side chains can be phosphorylated to yield phosphoserine (pSer) and phosphothreonine (pThr). Serine and threonine phosphorylation accounts for more than 97% of phosphate esterified to protein amino acids and is involved in several cellular signaling pathways (Plattner and Bibb, 2012).

pY signaling requires the presence of protein domains capable of binding specifically to amino acid sequences containing this residue. Among them, Src-homology 2 (SH2) domains are by far the most abundant and well characterized. Other notable examples of pY-recognizing domains are the PTB (Kavanaugh et al., 1995), C2 (Benes et al., 2005), HYB (Mukherjee et al., 2012) and pyruvate kinase M2 (Christofk et al., 2008) domains. In contrast, phosphorylation of serine and threonine side chains acts predominantly to induce conformational change, rather than mediating protein-protein interaction. Nevertheless, several modular domains (*e.g.* WW and FHA domains or 14-3-3 proteins) specifically recognize pSer and pThr marks (Yaffe and Elia, 2001).

**Fig. 1:**
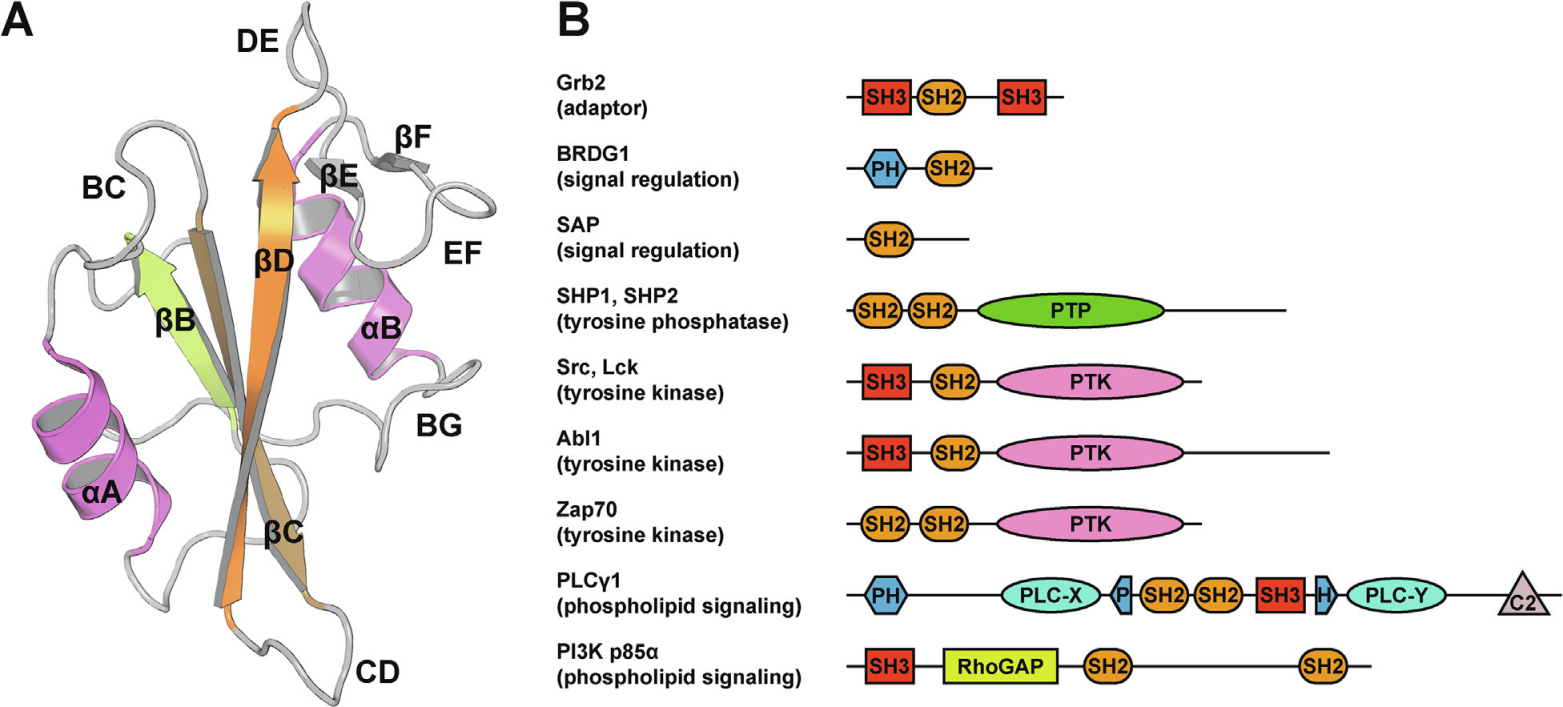
**General architecture of SH2 domains and their distribution in cellular proteins.** (A) The crystal structure of the SH2 domain of Lck (PDB entry 1LCJ) shows the highly conserved fold consisting of a central β-sheet core flanked by two α-helices. Minor variations in secondary structure elements have been reported in other SH2 domains, including short β-strands before the first α-helix (βA) and after the BG loop (βG). The naming of secondary structure elements and loops follows the convention of Eck et al. (1993). Secondary structure elements are color-coded (αA and αB in pink, βB in light green, βC and βD in orange, loops in grey) (B) Many of the SH2 domain-containing proteins referred in this text feature a modular composition and exert a wide variety of roles in the cell. Proteins with SH2 domains have also been implicated in chromatin remodeling, gene transcription, ubiquitination and cytoskeletal reorganization. For a more complete list, see Liu et al. (2006). SH3: Src-homology 3; PH: Pleckstrin homology; PTP: Protein tyrosine phosphatase; PTK: Protein tyrosine kinase. (For interpretation of the references to color in this figure legend, the reader is referred to the web version of this article.)

In the context of pY signaling, the SH2 module was originally discovered as a conserved subunit in the v-Fps/Fes tyrosine kinase (Sadowski et al., 1986). SH2 domains consist of approximately 100 amino acids with an invariant fold, in which a central three-stranded antiparallel β sheet, interspersed with shorter parallel β strands, is flanked by two α helices. The secondary structure elements are joined by loops, whose sequence and length are less conserved. Following the convention introduced by Eck and coworkers in 1993, the strands are named βA–βG, the helices are αA and αB, and the loops are named according to the letters of the structural elements that they join. Phosphopeptide residues are numbered based on their position relative to pY (… pY-2, pY-1, pY+1, pY+2 …) (Eck et al., 1993) (Fig. 1A). Approximately 120 different SH2 domains, distributed among more than a hundred different proteins, have been identified in the human genome (Liu et al., 2006), highlighting their importance in cell physiology (Fig. 1B). In agreement, it has been proposed that the pY-signaling machinery may have facilitated metazoan evolution (Shiu and Li, 2004).

By specifically recognizing pY motifs, SH2 domains allow precise spatial and temporal regulation of the proteins that contain the pY mark. A prominent example is the protein adapter Grb2 in the context of Ras/MAPK signaling, whose SH2 domain recognizes tyrosyl-phosphorylated sequences of the cytosolic domain of receptor tyrosine kinases (RTKs): this allows the localization of the guanine-nucleotide exchange factor SOS, which is bound to Grb2, in the proximity of Ras, ultimately leading to its activation (Lemmon and Schlessinger, 2010). Some SH2 domains regulate the activity of enzymes, such as Src-family kinases Src, Yes, Fyn and Lyn (Boggon and Eck, 2004), tyrosine kinase Abl (Hantschel et al., 2003), tyrosine phosphatases SHP1 and SHP2 (Hof et al., 1998), and phospholipase PLCγ1 (Bunney et al., 2012). In addition, a subset of catalytically dead members in the PTP and dual-specificity phosphatase (DSP) families recognize pY peptides through other pY recognition domains, such as the PKCθ C2 or the pyruvate kinase M2 (Hunter, 2009).

The functional importance of SH2 domains explains their involvement in several human diseases. For example, mutations that abnormally increase the affinity of the N-SH2 domain of SHP2 for pY-ligands or disrupt its regulatory function have been implicated in the pathogenesis of Noonan and LEOPARD syndromes (Tartaglia and Gelb, 2005), as well as several malignancies (Keilhack et al., 2005, Niihori et al., 2005, Higashi et al., 2002). Furthermore, mutations in the *SH2D1A* gene, which encodes for the SLAM-associated protein (SAP), consisting almost exclusively of one SH2 domain, lead to the X-linked lymphoproliferative syndrome (XLP) (Poy et al., 1999).

Precise spatial and temporal control of signaling cascades requires fine-tuning of both the thermodynamic and kinetic parameters of the binding events involved in the process. For example, high-affinity interactions are long-lived and may provide higher specificity for one selected target; however, they may also impair the ability to react to rapidly changing conditions (Pawson, 2004). Specificity may be even reduced by mutations that increase affinity, probably due to binding to ectopic motifs (Kaneko et al., 2012, Kessels et al., 2002, Zarrinpar et al., 2003). In agreement with this, the affinities of most SH2 domains for pY motifs are modest.

The question of how a relatively invariant fold such as the SH2 domain achieves the specificity and binding kinetics required for proper control of pY-dependent signaling has been the subject of intense investigation. Crystallographic structures and thermodynamic studies have been extensively used to discover the specificity code. Over time, the conventional view that SH2 domains achieve selectivity by making specific interactions with peptide residues pY+1, pY+2 and pY+3 has proven too simplistic. Nuclear magnetic resonance (NMR) studies of the internal dynamics of SH2 domains have revealed new and unexpected determinants of the specificity code. Furthermore, *in vivo*, where several signaling pathways compete with each other, non-equilibrium processes (i.e. the kinetics of binding) become relevant. In this review, we discuss structural, dynamic, thermodynamic and kinetic data available for SH2 domains in complex with pY motifs. The review is divided in four sections summarizing structural (1), thermodynamic (2), dynamic (3) and kinetic (4) data available on SH2–phosphopeptides complexes. We conclude that a full understanding of binding specificity and regulation of enzyme activity through pY-dependent signaling requires consideration of both structure and dynamics of the molecules involved, as well as non-equilibrium kinetic processes.

## 2 Structure

**Fig. 2:**
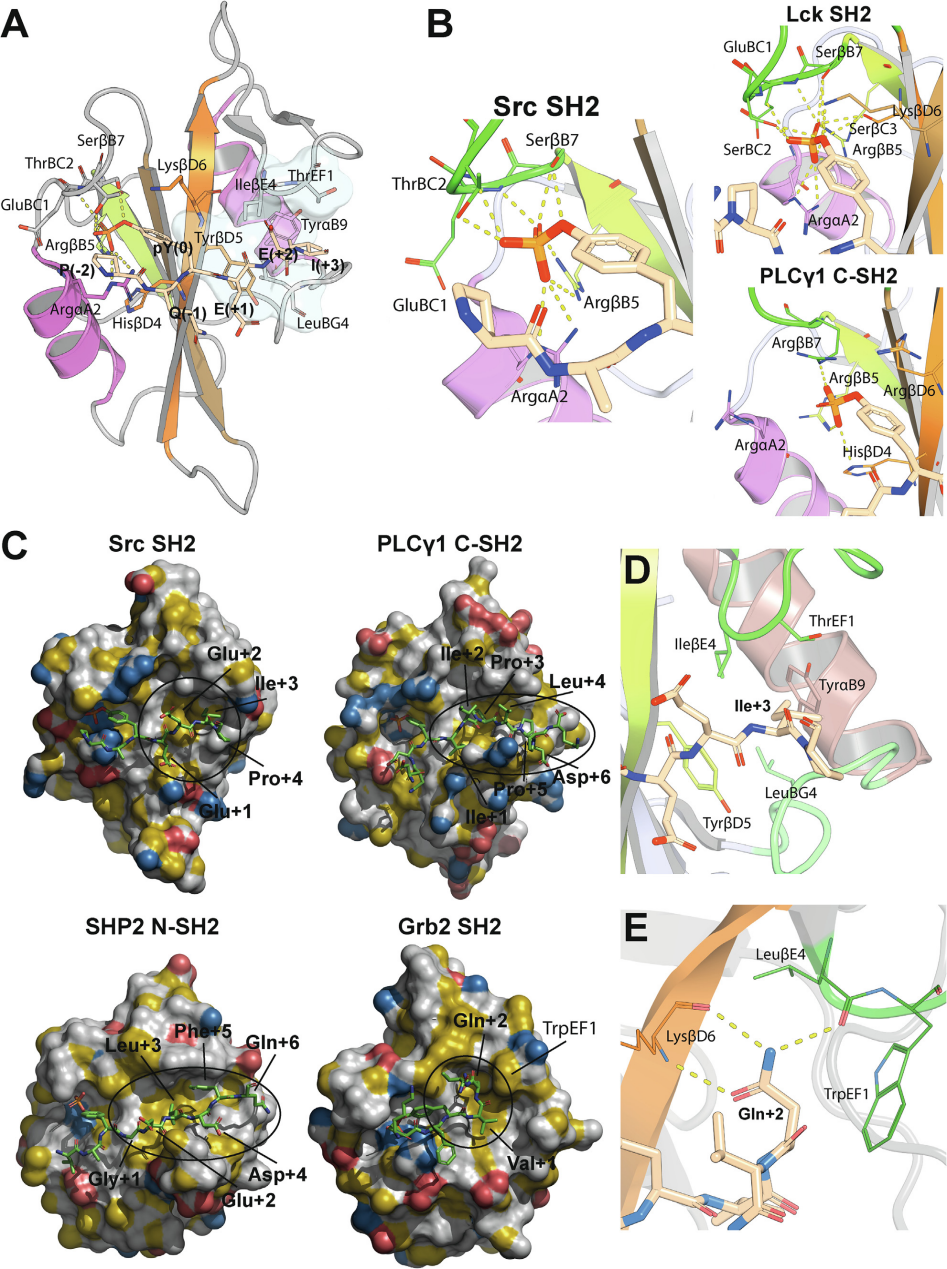
**Structural features of phosphopeptides recognition by SH2 domains.** (A) Overview of the two-pronged plug binding mode as seen in the structure of the Src SH2 domain in complex with the pYEEI peptide from the hamster polyomavirus middle-sized tumor antigen (PDB entry 1SPS). The color code is as in Fig. 1A (B) The binding mode of the phosphotyrosine moiety is remarkably conserved among SH2 domains, as shown here by the Src (left, PDB entry 1SPS) and Lck (middle, PDB entry 1LCJ) SH2 domains in complex with the pYEEI peptide from the hamster polyomavirus middle-sized tumor antigen, and the PLCγ1 C-SH2 domain in complex with a phosphopeptide derived from PDGFR (right, PDB entry 2PLD). Specifically, a universally conserved arginine (ArgβB5) provides a bidentate hydrogen bond to the phosphate group, corroborated by several amino acids from BC, αA, βC and βD, which show a greater degree of variability. (C) The “specificity pocket” of Src SH2, lined by hydrophobic amino acids, is responsible for the recognition of phosphopeptide residue pY+3 (Ile in the pYEEI peptide), providing specificity to the interaction (PDB entry 1SPS, two-pronged plug). Some SH2 domains recognize phosphopeptides in a different manner from the “two-pronged plug” binding mode typical for SH2 domains of the Src-family kinases; for example, PLCγ1 C-SH2 recognizes phosphopeptide residues up to pY+5/pY+6 (PDB entry 2PLD), SHP2 N-SH2 engages in contacts with pY+5 (PDB entry 6ROY), while the presence of a bulky TrpEF1 in Grb2 SH2 forces the peptide to adopt a β-turn conformation (PDB entry 1TZE). Color coding according to the YRB method (Hagemans et al., 2015) (blue: nitrogens carrying positive charges, red: oxygens carrying negative charges, yellow: hydrocarbon groups without polar substitutions.) (D) Zoom of the specificity pocket of Src SH2 in complex with the pYEEI peptide demonstrating the interaction between the protein TyrβD5 and the peptide Ile(pY+3). (E) In addition to forcing the phosphopeptide to adopt a β-turn conformation, TrpEF1 of Grb2 imposes the strict requirement of a glutamine at position pY+2 in phosphopeptides that bind this domain, since the side-chain amide group of this residue can make three hydrogen bonds with LysβD6 and LeuβE4 (PDB entry 1TZE). In panels B-E, the color code is as in Fig. 1A with the exception that the loops in contact with the phosphopeptide are in green. (For interpretation of the references to color in this figure legend, the reader is referred to the web version of this article.)

A large number of high-resolution structures are available for SH2–phosphopeptide complexes. The vast majority of SH2 domains binds to phosphopeptides in a canonical, two-pronged way (Fig. 2A): the peptide adopts an extended conformation, perpendicular to the central β sheet, with the pY hosted in a groove lined by βB, βC, βD, αA and the BC loop and held in place by electrostatic interactions and hydrogen bonds to the phosphate group (Eck et al., 1993). A second binding site, which is supposed to provide specificity to the interactions with phosphopeptides, is built by a largely hydrophobic pocket delimited by CD, DE, EF, BG, βD and αB, and accommodates peptide residues C-terminal to the pY. Despite substantial variations in the amino acid sequence of its surrounding loops, the structure of this pocket (termed the “specificity pocket”) is evolutionarily conserved (Liu et al., 2011).

The binding mode to the pY is similar in all SH2 domains (Waksman et al., 1992) (Fig. 2B). A universally conserved arginine (ArgβB5), whose side chain is buried from the solvent both in the free and pY-bound forms, contributes a bidentate salt bridge to two oxygen atoms of the phosphate group. Mutation of this residue abrogates pY binding both *in vitro* (Bradshaw et al., 1999) and *in vivo* (Bibbins et al., 1993). Other positively charged residues contribute to the stabilization of the pY, although they are less critical and may not be present in all SH2 domains (ArgαA2 and LysβD6 in Src SH2). For example, ArgαA2 makes a guanidino-aromatic interaction with the pY aromatic ring in Src-family SH2 domains, but is absent in SHP2 N-SH2, where it is replaced by a glycine (Lee et al., 1994, Huyer et al., 1995, Huyer and Ramachandran, 1998). In Src SH2 both SerβB7 and ThrBC2 make hydrogen bonds with the pY phosphate, but their removal has only a marginal effect on pY affinity (Bradshaw and Waksman, 2002) (Fig. 2B). Interestingly, Src SH2 has a unique cysteine in the pY-binding pocket (CysβC3): mutation of this residue to serine improves binding affinity to phosphopeptides by 4- to 8-fold (Bradshaw et al., 1999). In summary, binding of pY to SH2 domains is based on electrostatic interactions, as confirmed by the dependence of the binding affinities on pH (Bradshaw and Waksman, 1998, Singer and Forman-Kay, 1997) as well as the nature and concentration of the buffer salt (Grucza et al., 2000).

The binding mode to peptide residues other than pY is more variable and has important consequences for the specificity of the different classes of SH2 domains. Most SH2 domains, whose prototype is the Src SH2 domain, bind phosphopeptides in the canonical or “two-pronged plug” fashion (Fig. 2A): in addition to the pY-binding site, the “specificity pocket” buries the side chain of the pY+3 residue (Waksman et al., 1993) (Fig. 2C). In Src SH2 the hydrophobic residues forming this (+3) pocket are TyrβD5, LeuBG4, IleβE4, ThrEF1 and TyrαB9 (Fig. 2C). Residue βD5 has a crucial role in determining the specificity for the pY+3 amino acid (Songyang et al., 1993, Huang et al., 2008). Src SH2 and other Src-family kinase SH2 domains have an aromatic residue at βD5, which dictates the requirement for a hydrophobic side chain at pY+3, with Ile(+3), Leu(+3) and Val(+3) being the most favored amino acids. The other protein–peptide interactions are generally less tight and/or mediated by water molecules (Bradshaw and Waksman, 2002).

A few SH2 domains, which have an aliphatic residue at βD5 bind as a two-pronged plug but require residues other than Ile, Leu or Val at pY+3 (Huang et al., 2008)). For example, the SH2 domains of the p85 subunit of the phosphoinositide-3-kinase (PI3K) bind consensus sequences pY-M-X-M, where X is any amino acid: the presence of IleβD5 (p85 N-SH2) or CysβD5 (p85 C-SH2) instead of TyrβD5 (as in Src SH2) makes the (+3) binding pocket narrower and deeper and at the same time generates a second hydrophobic patch that accommodates a hydrophobic residue at pY+1 (Breeze et al., 1996, Nolte et al., 1996).

Besides the two-pronged plug model, several SH2 domains bind peptide residues beyond pY in different ways. The C-SH2 domain of PLCγ1, for example, has been shown to interact with phosphopeptide residues up to pY+5/pY+6 by means of a mostly hydrophobic, extended, open groove (Pascal et al., 1994) (Fig. 2C). A similar extended hydrophobic pocket is present in the SH2 domains of SHP2 and allows the phenylalanine pY+5 of IRS1- or PD-1-derived peptides to bind between the EF and BG loops (Lee et al., 1994, Marasco et al., 2020) (Fig. 2C, D). SHP2 N-SH2 is rather unique in its versatility and binding modes: in addition to its unusual requirement for a hydrophobic residue at pY-2, dictated by the presence of a glycine at αA2, this domain is capable of binding phosphopeptides by recognizing only residues N-terminal to the pY (Qin et al., 2005), two phosphopeptides at the same time (Zhang et al., 2011), or a phosphopeptide in a reverse direction (Wang et al., 2018).

BRDG1 SH2 is another unusual SH2 domain, which has a defined binding pocket for pY+4 and requires a leucine at this position. The (+4) binding pocket resembles a pentagon basket and is structurally conserved in all SH2 domains; however, except for BRDG1 SH2 and a few other related SH2 domains, this pocket is inaccessible due to an intramolecular interaction with a leucine or isoleucine of the BG loop (Kaneko et al., 2010). The accessibility of the (+4) pocket is encoded in the length and composition of the EF and BG loops: changing these two features was demonstrated to alter the accessibility of the pocket and thus the specificity of the SH2 domain (Kaneko et al., 2010, Liu et al., 2019).

The Grb2 SH2 domain displays the most unconventional binding mode to phosphopeptides (Fig. 2C). Despite having an aromatic residue at βD5, it does not bind to the same consensus sequences as Src-family kinase SH2 domains but has a very strong selectivity for peptides with asparagine at pY+2. This feature is explained by the presence of a Trp residue (TrpEF1) that occludes the (+3) binding pocket and forces the peptide backbone to adopt a β-turn conformation (Rahuel et al., 1996). In this unusual conformation, the amide group of the asparagine provides the hydrogen bonds necessary to stabilize the complex (Fig. 2E).

Finally, the SLAM-associated protein (SAP) lifts the paradigm that pY is mandatory for the interaction. SAP consists almost exclusively of an SH2 domain, which recognizes peptide sequences longer than usual and makes specific interactions with peptide residues pY–3 and pY+2, leading to a 3-pronged binding mode (Morra et al., 2001, Hwang et al., 2002). Because of these additional contact points, SAP can bind non-phosphorylated peptide sequences, although with lower affinity than in the presence of pY. Likely, this peculiar binding mode is related to the function of SAP: first, it can prevent phosphorylation of tyrosine-containing sequences by binding to their non-phosphorylated state; second, the tight, three-pronged binding to pY-containing peptides can shield them from other SH2-docking sites or from phosphatase action (Poy et al., 1999, Sayos et al., 1998, Peled et al., 2018).

In conclusion, despite the structural and sequence conservation of SH2 domains, small differences in the amino acid sequence are able to generate a wide diversity of binding modes that likely serves the purpose of optimizing the thermodynamic parameters of the binding event for its specific function and regulation.

## 3 Thermodynamics

The affinities of SH2 domains for cognate phosphopeptides span around three orders of magnitude. A few examples of very high-affinity SH2–phosphopeptide complexes have been reported in the literature, such as the SHP2 N-SH2 domain in complex with a phosphopeptide derived from IRS-1 (dissociation constant *K_D_* = 14 nM, (Sugimoto et al., 1994)); however, most SH2 domains interact with phosphopeptides with a *K_D_* in the 100 nM – 10 µM range (Ladbury and Arold, 2011). The question of whether such moderate affinities serve a biological role has been recently addressed by Kaneko and coworkers (Kaneko et al., 2012), who designed mutant constructs of the Fyn and Src SH2 domains capable of binding pY ligands with substantially higher affinities than the wild type proteins. Interestingly, when these “pY superbinders” were expressed in HEK293 cells, they blocked EGFR signaling and inhibited anchorage-independent cell proliferation, indicating that tight binding of SH2 domains to pY ligands is detrimental for function. It was proposed that moderate affinities serve the purpose of maintaining the rapid dynamic control required in pY signaling cascades (Pawson, 2004).

Early studies investigated the energetics of the binding of pY-ligands to SH2 domains by isothermal titration calorimetry (ITC) and showed that in most cases the binding is driven by a large favorable enthalpy contribution (ΔH) and a smaller, but also favorable, entropic contribution (TΔS) (*e.g.* ΔH = −7.7 kcal/mol, TΔS = 1.5 kcal/mol for Src SH2 in complex with pYEEI (Bradshaw et al., 1999); ΔH = −7.94 kcal/mol, TΔS = 1.18 kcal/mol for Grb2 SH2 in complex with Shc-pY317 (McNemar et al., 1997)). On average, binding of the pY moiety alone contributes approximately half of the total free energy of binding (Bradshaw et al., 1999). This allows very efficient and specific recognition of phosphorylated targets and depletes almost completely the binding to non-phosphorylated peptides; however, it also leaves little room for generating specificity through interactions with peptide residues N- or C-terminal to the pY (Ladbury and Arold, 2011).

**Fig. 3:**
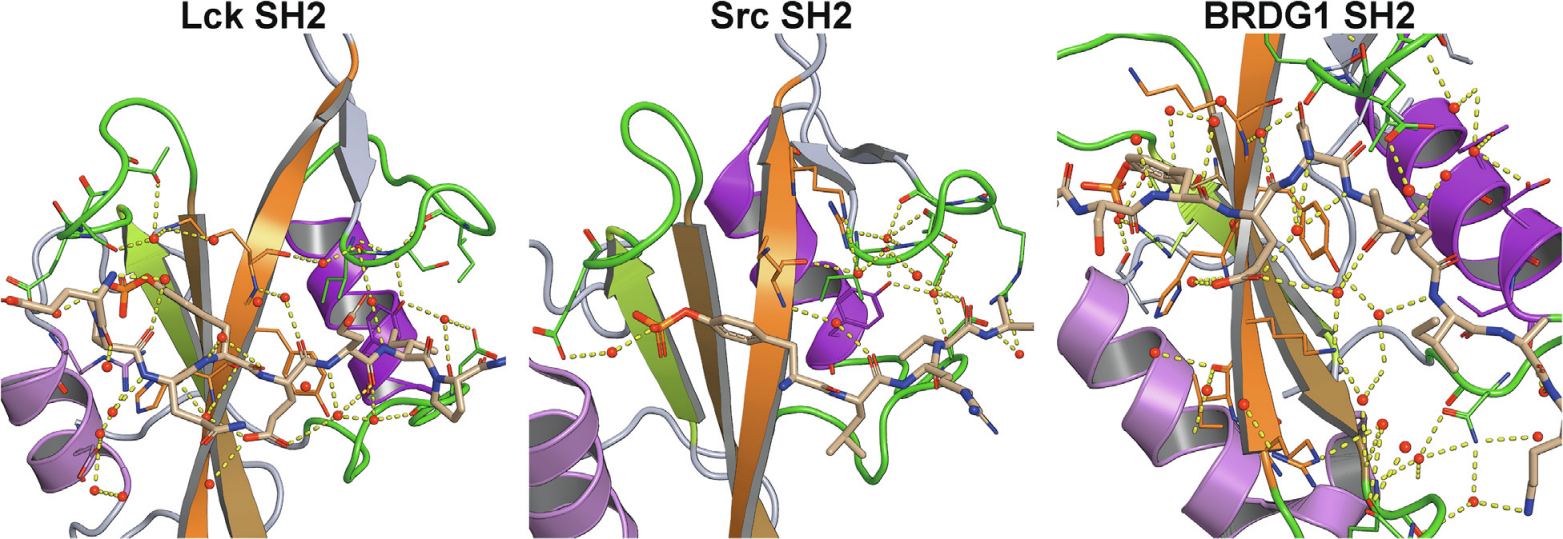
**Structural water molecules mediating protein–peptide interactions.** High-resolution crystal structures of three different SH2 domains, in which the structural waters located within 6 Å of the phosphopeptide are shown as red spheres. The binding pockets of most SH2 domains have been shown to be hydrated: the binding of the water molecules should contribute a negative entropy term to the free energy of binding. PDB entries: 1LCJ (Lck SH2), 1SHB (Src SH2), 3MAZ (BRDG1 SH2). The color code is as in Fig. 1A with the exception that the loops in contact with the phosphopeptide are in green. (For interpretation of the references to color in this figure legend, the reader is referred to the web version of this article.)

In contrast to phosphopeptides, whose binding is driven by enthalpy, the binding of isolated phosphotyrosine is entropically driven. The pY binding pocket is engaged in a network of hydrogen bonds to water molecules in the protein apo state; these water molecules are competed out by the phosphotyrosine, which forms a new network of hydrogen bonds with the SH2 residues. The release of bound water accounts for the increase in entropy (Ladbury and Arold, 2011). Interestingly, binding of isolated pY can cause perturbation of NMR chemical shifts of SH2 domains also in regions distant from the binding pocket, as revealed by solution NMR for the N-SH2 domain of SHP2 (data not shown), suggesting coupling between the pY binding pocket and other secondary structure elements. In general, binding of the pY moiety provides most of the favorable entropic change, while the binding of the other peptide residues is enthalpically favorable and associated with a negative entropy change. One contribution to the negative entropy change may be provided by structural water molecules, which have been found to mediate protein–peptide interactions in several high-resolution X-ray structures of SH2–phosphopeptide complexes (*e.g.* Lck SH2–phosphopeptide, PDB entry 1LCJ (Eck et al., 1993); Src SH2–phosphopeptide, PDB entry 1SHB (Waksman et al., 1992); BRDG1 SH2–phosphopeptide, PDB entry 3MAZ (Kaneko et al., 2010)) (Waksman et al., 1993) (Fig. 3).

In a pioneering study, Songyang and coworkers used a phosphopeptide library and affinity purification to unravel the contribution of residues C-terminal to the phosphotyrosine (pY+1, pY+2 and pY+3) to the binding specificity of distinct SH2 domains (Songyang et al., 1993). Beads loaded with a single SH2 domain were incubated with the phosphopeptide library and bound peptide sequences were isolated and quantified. The authors also proposed a classification system of SH2 domains based on the identity of the residue at position βD5, which forms a platform for the peptide backbone and has a direct interaction with pY+1: a moderate correlation was found between SH2 domain families and preferred binding motifs. The classification of SH2 domains was updated later, resulting in three major groups, each of which was further divided into several subgroups, accounting for fine-tuning of specificity (Huang et al., 2008). In addition, the same authors found that some SH2 domains also discriminate for residues N-terminal to the phosphotyrosine.

Although very informative, all these studies lack a proper evaluation of the difference in binding affinity between interactions to “specific” and “non-specific” motifs (Ladbury and Arold, 2011). For those domains where thermodynamic parameters were measured, the results are apparently in contradiction with the conventional view that residues pY+1 to pY+3 strongly discriminate between specific and non-specific interactions. For example, the pYEEI motif, which was determined to be the best binding sequence for the Src SH2 domain, shows only up to two orders of magnitude better affinity when compared to two unrelated phosphopeptides (Bradshaw and Waksman, 2002, Bradshaw et al., 1998). Furthermore, conservative mutations at positions pY+1, pY+2 and pY+3 diminished affinity of only threefold, while mutations to alanine were necessary to cause a reduction in binding affinity of one to two orders of magnitude (Bradshaw and Waksman, 1999). This is somewhat surprising, considering that all three residues immediately after pY are involved in an extensive network of interactions with the protein. The strongest decrease in affinity was obtained when Glu(+1) was replaced with a glycine (50-fold) (Bradshaw and Waksman, 1999), as well as upon mutation of the interacting protein residue TyrβD5 (Bradshaw et al., 2000), indicating that the Glu(+1)-Hβ–TyrβD5-aromatic contact is energetically important in complexes involving SH2 domains of the Src family (Bradshaw and Waksman, 1999) (Fig. 2D). Interestingly, mutation of Ile(+3) to alanine did not have a large impact, even though the isoleucine side chain is deeply accommodated in the hydrophobic specificity pocket (Fig. 2D). Similarly, mild effects on affinity were measured upon mutation of Src SH2 residues that interact with the EEI motif (Bradshaw et al., 2000).

**Fig. 4:**
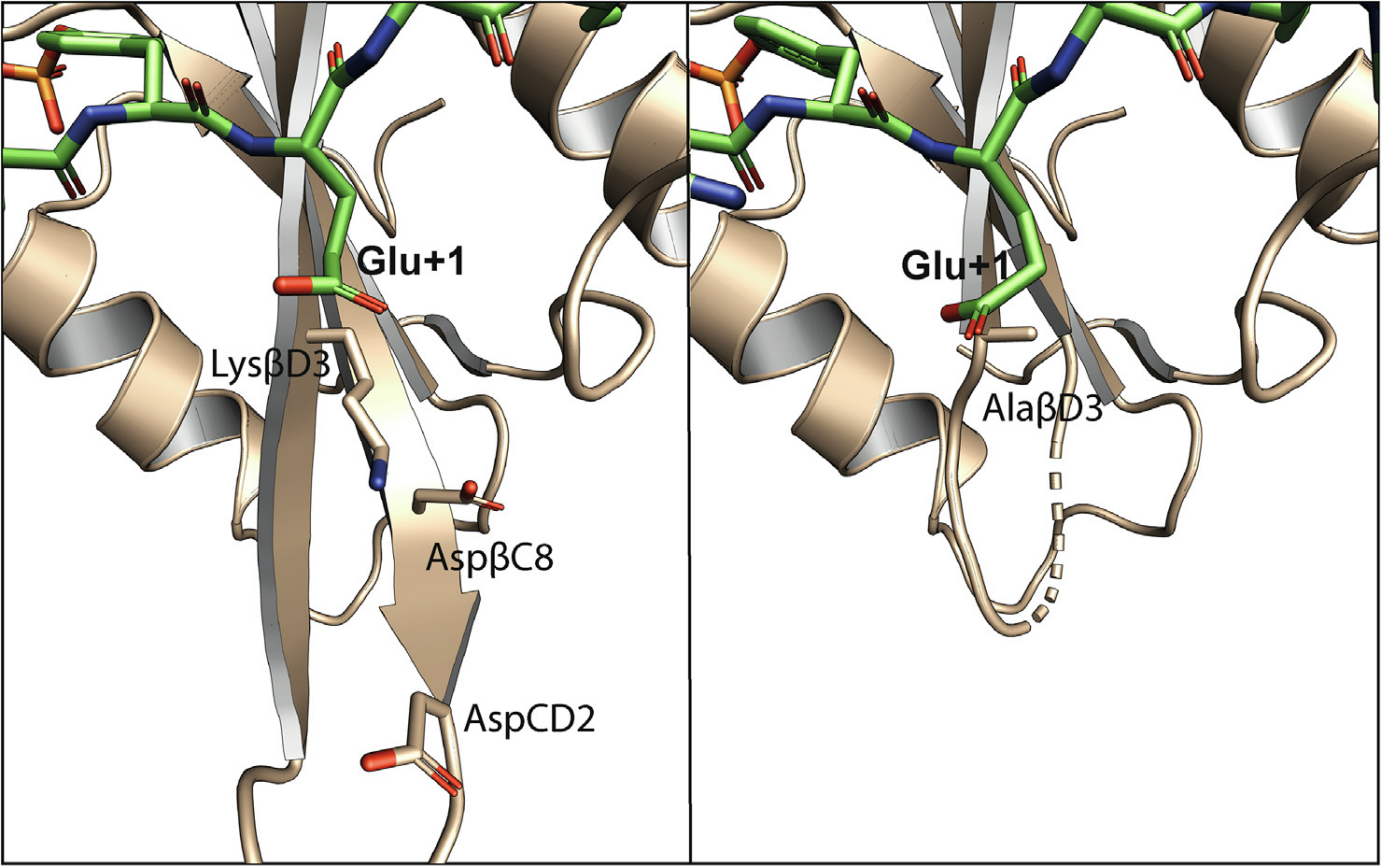
**Structural consequences of mutations on the binding surface of the pY+1 amino acid.** Comparison of the crystal structures of wild-type Src SH2 (left, PDB entry 1SPS) and triple-mutant Src SH2 (right, LysβD3Ala-AspCD2Ala-AspβC8Ala, PDB entry 1KC2) in complex with the pYEEI phosphopeptids. Rreplacement of LysβD3 with an alanine reduces the affinity of the SH2 domain for the phosphopeptide because the negative charges of AspβC8 and AspCD2 are no longer shielded. Notably, the CD loop is disordered in the triple mutant SH2 domain.

The analysis of thermodynamic data of sequentially mutated peptides did not show any significant coupling between peptide residues (Ladbury and Arold, 2011). Similarly, double mutant cycle experiments failed to find any coupling between the Src SH2 residues and the phosphopeptide residues C-terminal to pY (Bradshaw and Waksman, 1999, Lubman and Waksman, 2002), with the significant exception of the interaction between Glu(+1) and LysβD3. The LysβD3Ala mutation reduced the binding affinity for pYEEI, albeit not as much as TyrβD5Ala. Nearly full binding affinity could be restored when Glu(+1) was also mutated to alanine. To explain this, Lubman and Waksman showed that the energetic coupling between LysβD3 and Glu(+1) is due to the presence of AspβC8 and AspCD2, whose negative potential is neutralized by LysβD3 and instead interferes with Glu(+1) binding in the LysβD3Ala mutant (Fig. 4) (Lubman and Waksman, 2002). The authors proposed that selectivity at pY+1 is not due to a single interaction, but rather to the entire functional patch formed by LysβD3, AspβC8, AspCD2 and ArgAB6; the perturbation of this patch can also influence the binding of distant residues (pY-1 and pY-2) (Lubman and Waksman, 2002).

Similar results were found for SH2 domains in proteins outside the Src family. The p85 N-SH2 domain, which was originally shown to be selective for Met(+3) (Songyang et al., 1993), binds randomized peptides only 60-fold weaker than the optimal motif pYMXM (Piccione et al., 1993); a more prominent loss in affinity was measured only upon mutation of either of the two methionines to glycine (Günther et al., 1996). For the C-SH2 domain of PLCγ1, only residue pY+1 was found to contribute a large binding energy, although this domain interacts with peptide residues up to pY+6 (Pascal et al., 1996; Kay et al., 1998). Uniquely, the SH2 domain of Grb2 has a very strong preference for asparagine at pY+2 (Songyang et al., 1993) and the Asn(+2)Ala mutation causes a 1000-fold decrease in affinity (McNemar et al., 1997).

Several signaling proteins have two or more different SH2 domains arranged in tandem, which leads to far greater specificity than in the case of individual SH2 domains. This is due both to the multiplicative effect of the intrinsic binding specificity of each SH2 domain and to the orientation and spacing requirements imposed by the tandem arrangement on the pY sites (Ottinger et al., 1998). Ottinger et al, who used surface plasmon resonance (SPR) to study the interaction of the tandem SH2 domains of SHP2, PI3K p85, ZAP70/Syk and PLCγ1 with several doubly phosphorylated tyrosine activation motifs, revealed that each tandem SH2 binds the specific cognate ligand with very high affinity (*K_D_* in the pM–nM range); furthermore, the specificity measured in competition experiments was found to be much higher than for isolated SH2 domains with values in the range 1000-fold –10,000-fold (Ottinger et al., 1998).

All these data indicate that the specificity of the SH2 domains for their cognate phosphopeptide sequence is mild and allows for crosstalk between different signaling pathways. Most of the proteins involved in signaling contain multiple SH2 domains, sometimes accompanied by other domains recognizing other moieties than phosphotyrosine. Likely, it is the combination of these recognition events that builds up specificity while allowing for a granular, stepwise regulation of cellular response.

Another take-home message from the thermodynamic data is that binding affinities cannot be described as a sum of point interactions between the ligand and the receptor protein: often, to explain changes in binding energy upon mutations, one needs to invoke a network of interactions, where the perturbation of a node results in subtle structural rearrangements throughout the network. In addition, entropic contributions to the binding affinities, in form of protein and peptide dynamics, may play a role as well. These will be discussed in the following section.

## 4 Dynamics

Besides the enthalpic contributions and the entropy increase due to the release of bound water, changes in the internal dynamics of the binding partners may modulate protein–peptide affinities and specificity. NMR spectroscopy is the major technique capable of measuring differences in the dynamics of the unbound and bound states in a large range of time scales.

In a pioneering study, Farrow et al. characterized the backbone dynamics of unbound and bound states of the PLCγ1 C-SH2 domain in the ps–ns time scale (Farrow et al., 1994). They found that phosphopeptide binding is not associated with a reduction of protein mobility at the binding site, as it had been previously measured for other protein–ligand complexes (Akke et al., 1993, Nicholson et al., 1992). In general, PLCγ1 C-SH2 showed little high-frequency motions in its unbound state; peptide binding was associated with increased order parameters (i.e. greater rigidity) of only a few residues, such as ArgβB7 in the pY binding pocket, while several other residues became more disordered instead (Farrow et al., 1994). Measurements of slower dynamics, corresponding to conformational exchange processes in the μs–ms time scale (quantified through the parameter *R_ex_*), showed no significant change upon peptide binding either (Farrow et al., 1994). A follow-up study measured the dynamics of peptide-bound PLCγ1 C-SH2 arginine side chains in the pY binding pocket (Pascal et al., 1995). This SH2 domain features an unusually high number of arginines, four in total, that can potentially interact with the phosphate group, while the pY binding pockets of other SH2 domains, such as Src SH2 and SHP2 N-SH2, contain either two or one conserved arginines, respectively. Three of the four arginines of PLCγ1 C-SH2 (ArgαA2, ArgβB5 and ArgβB7) are inaccessible to solvent: they showed limited fast motions but considerable line broadening due to μs–ms time scale dynamics. Conversely, the fourth arginine (ArgβD6) was found to have considerable high-frequency motions.

A major contribution to understanding the change of protein dynamics upon peptide binding and its impact on binding activity and selectivity was made by the Kay laboratory with a study on sidechain methyl-group motions of PLCγ1 C-SH2 in the ps–ns time scale (Kay et al., 1996). The pY binding pocket was found to become more rigid upon peptide binding, while the hydrophobic sidechains of the specificity pocket remained mobile, indicating a relaxed side-chain packing in this region. Later, Kay and coworkers used this dynamic behavior to explain the apparent conflict between structural data, which indicate that PLCγ1 C-SH2 engages in extensive contacts with the phosphopeptide (Pascal et al., 1994), and binding studies that show little influence of peptide mutations on the binding affinities (Kay et al., 1998). They proposed that the presence of large-amplitude motions in the hydrophobic surface of the specificity pocket, together with the very steep distance-dependence of the van der Waals energy, lead to weaker interactions in this region and thus greater permissivity (Kay et al., 1996). In contrast, the specificity pocket of the SHP2 N-SH2 domain bound to an IRS1-derived phosphopeptide is less flexible and the truncation of the phosphopeptide C-terminal residues leads to a more dramatic decrease in affinity than in the case of PLCγ1 C-SH2 (Kay et al., 1998). Thus, a correlation seems to exist between ps–ns dynamics and binding affinity, which can explain different specificity properties of two structurally very similar domains. In the case of PLCγ1 C-SH2, the high-frequency motions of the hydrophobic residues in the specificity pocket may serve the purpose of reducing the affinity for the phosphopeptide and maintain reasonably fast dissociation rates. This may become necessary due to the presence of four arginines in the pY binding pocket of PLCγ1 C-SH2, which provide a far stronger electrostatic interaction with the phosphotyrosine than the single arginine in SHP2 N-SH2.

More recently, Finerty et al. studied the backbone and sidechain dynamics of a phosphopeptide (DNDpYIIPLPDPK) derived from PDGFR bound to PLCγ1 C-SH2 (Finerty et al., 2005). Amide ^15^N order parameters identify pY, Ile(+1) and Ile(+2) as the most rigid amino acids. Contrarily, methyl groups order parameters are all low, except for the Ile(+1) γ2, thus mirroring the high level of dynamics of the protein sidechains. The authors conclude that the intermolecular interface C-terminal of pY is characterized by a high level of sidechain conformational exchange, which modulates the affinity between the ligand and the protein (Finerty et al., 2005).

Notably, PLCγ1 C-SH2 exists in solution in equilibrium between monomeric and dimeric species, even at relatively low protein concentrations (Farrow et al., 1994). The dimerization seems to be mediated by the large hydrophobic specificity pocket, which becomes shielded by peptide binding, thus recapitulating the shift of the equilibrium towards the monomeric state upon complex formation. A tendency to dimerize has been observed also for SH2 domains of other proteins, including fyn (Pintar et al., 1996), hck (Zhang et al., 1998) and SAP (Finerty et al., 2002), although in these cases peptide binding does not lead to complete dissociation of the dimers.

In summary, protein internal dynamics may have considerable influence on the strength of intermolecular interactions, finely tuning the balance between specificity and permittivity as well as the capability to respond to signaling.

## 5 Kinetics

The binding of signaling peptides to their receptors depends not only on thermodynamic parameters, but also on the kinetics of the intermolecular interactions. Kinetic parameters are particularly relevant in the presence of competitive binding partners with variable local concentrations, as usually found in the cellular environment. Thus, *in-vivo* the association and dissociation constants of the molecular complexes involved in the signaling processes (*k*_on_ and *k*_off_) cannot be neglected.

Early studies used surface plasmon resonance (SPR) to measure the kinetic parameters of SH2 domains binding to phosphopeptides in isolated systems (Felder et al., 1993, Panayotou et al., 1993). Both studies found very high *k*_on_ rates (between 3 × 10^7^ to 40 × 10^7^ M^−1^ s^−1^ for the SH2 domains of p85 binding PDGFR-derived phosphopeptides (Felder et al., 1993) and 3.34 × 10^6^ M^−1^ s^−1^ for p85 N-SH2 binding a peptide representing the pY751 site of PDGFR (Panayotou et al., 1993)). The dissociation constants ranged from 0.11 to 0.19 s^−1^. Surprisingly, the calculated *K_D_* values were in the range 0.3–3 nM, namely two orders of magnitude smaller than those derived by ITC in other studies. This discrepancy suggested a problem in the experimental setup, which was later found to lay in the dimerization of the GST tag attached to the SH2 domains leading to artefactual avidity effects in the SPR experiments (Ladbury et al., 1995).

NMR studies, performing line shape analysis of the p85 N-SH2 domain signals upon titration of a peptide representing the pY751 site of PDGFR, delivered much lower values for the dissociation constant: the binding event was found to consist of two steps, presumably a first encounter step followed by a conformational change step, with *k*_off_ of 100 and 10 s^−1^, respectively (Hensmann et al., 1994). Thus, even *in vitro*, the kinetic parameters of binding of phosphopeptides to SH2 domains, remain controversial.

The *in vivo* kinetics of pY-dependent signaling events has been addressed in more recent publications. Oh et al used total internal reflection microscopy to show that the dwell time of Grb2 SH2 near the plasma membrane in the context of EGFR signaling is one order of magnitude longer than the *k*_off_ rate measured with isolated Grb2 and membrane-embedded EGFR using single-molecule fluorescence (Morimatsu et al., 2007) (0.52 s^−1^ *in vivo* versus 8 s^−1^ in the isolated system) (Oh et al., 2012). This discrepancy was shown to result from repeated rebinding of the Grb2 SH2 to multiple phosphotyrosine motifs *in vivo*, which is facilitated by receptor clustering. The phenomenon of rebinding in the presence of receptor clustering may also explain why deletion of a single high-affinity phosphotyrosine site on a receptor tyrosine kinase has only moderate effects on signaling (Oh et al., 2012). When measuring the dwell times of tandem SH2 domains in the presence of multivalent phosphopeptides, Oh et al found a more complex behavior, which they explained invoking the existence of both monovalent and divalent binding events; in divalent binding events, the data indicated that the binding of the first SH2 domain of the tandem to the first pY site occurs much more rapidly than the binding of the second SH2 domain to the second pY site (Oh et al., 2012). This result is in agreement with our recent work on the interaction between the tyrosine-phosphatase SHP2, which contains two SH2 domains in tandem, and a divalent peptide containing both the ITIM and ITSM pY sites of the receptor PD-1: our data demonstrated that the second binding event is slowed down by a conformational change in the linker joining the two SH2 domains, which is required to adopt a conformation compatible with the binding of the second pY site (Marasco et al., 2020).

In general, to understand the mechanisms of the fine regulation of signaling events by local concentration and receptor clustering, as well as the interplay between multiple signaling pathways, more kinetic data need to become available both *in vitro* and *in vivo*.

## 6 Conclusions

The complexity of phosphotyrosine-dependent signaling is impressive, especially since most of the pY recognizing domains belong to the family of SH2 domains with a well-conserved fold and pY recognition mode. The versatility in the function, specificity and regulatory capabilities of the SH2–phosphopeptide complexes relies on different mechanisms. First, while all SH2 domains recognize the pY mark with high specificity, the nature and strength of the interactions with the amino acids surrounding the pY are variable. For each complex, these interactions are tuned to yield sufficient specificity without compromising turn over. The tuning is achieved by a delicate equilibrium of enthalpic and entropic contributions to the binding energy, including internal motions of the SH2 domain sidechains. Second, the kinetics of the SH2–phosphopeptide interactions are likely to play an important role in the regulation of signaling events *in vivo*, where each phosphopeptide competes with others for SH2 domain binding, and local concentrations of individual molecules can vary over a wide range. Unfortunately, the kinetics of SH2–phosphopeptide complexes have been poorly studied both *in vitro* and *in vivo*. Third, nature uses multivalent binding events, relying on tandem SH2 domains and amino acid sequences containing multiple phosphorylation sites, to generate a large arsenal of both thermodynamic and kinetic parameters that can be tuned as needed to yield specific function and rapid response to changing environmental conditions. All in all, static structures of isolated SH2 domains bound to their cognate peptides are generally unable to explain function and regulation of the cellular processes in which they are involved; instead, they are the starting point for an exciting discovery tour in the world of thermodynamics and kinetics of multivalent and competing binding events.

## Declaration of Competing Interest

The authors declare that they have no known competing financial interests or personal relationships that could have appeared to influence the work reported in this paper.
